# Supplementary material for: Complementary and Alternative Medicines Used by Middle-Aged to Older Taiwanese Adults to Cope with Stress during the COVID-19 Pandemic: A Cross-Sectional Survey
Source: Healthcare (Basel). 2022 Nov 10;10(11):2250. doi: 10.3390/healthcare10112250 (PMC9690493; doi:10.3390/healthcare10112250)
Supplement: Supplementary file 1 [file healthcare-10-02250-s001.zip › healthcare-2020445-supplementary.pdf]

Supplement A. Questionnaire

CAM used to cope with stress during the COVID-19 pandemic period

I. Demographic characteristics

1. Gender: ☐ Female ☐ Male
2. Date of Birth: _______ Year _______ Month ______ Date
3. Height: _________ cm; Weight:_________ kg
4. Marital status: ☐ Single ☐ Married ☐ Divorced ☐ Widow ☐ Other
5. Number of children: ☐ 0 ☐ 1 ☐ 2 ☐ 3 or above
6. Education level: ☐ Elementary school or below ☐ Middle school ☐ High school ☐ College or University ☐ Master or above
7. Occupation: ☐ No or Retired ☐ Teacher or Public employee ☐ Salesmen or Attendance ☐ Skilled or Professional ☐ Other
8. Religion: ☐ No ☐ Buddhism ☐ Taoism ☐ Christian or Other

II. Life Style (Please choose the appropriate answer within the past half year)

1. Exercise: ☐ No ☐ Irregularly ☐ Regularly
2. Smoking: ☐ No ☐ Irregularly ☐ Regularly
3. Alcohol use: ☐ No ☐ Irregularly ☐ Regularly
4. Betel nut chewing: ☐ No ☐ Irregularly ☐ Regularly
5. Drinking coffee: ☐ No ☐ Irregularly ☐ Regularly
6. Drinking functional beverage: ☐ No ☐ Irregularly ☐ Regularly
7. Drinking milk: ☐ No ☐ Irregularly ☐ Regularly
8. Vegetarian diets: ☐ No ☐ Irregularly ☐ Regularly

III. Health conditions and medication use within the past half year (Please choose the appropriate answer/s within the past half year)

1. Perceived health status: ☐ Very poor ☐ Poor ☐ Fair ☐ Good ☐ Very Good
2. Have you been diagnosed or with one of the following chronic diseases?
   ☐ Hypertension ☐ Hyperlipidemia ☐ Diabetes ☐ Sleep disorders
   ☐ Upset stomach ☐ Presbyopia ☐ Myopia ☐ Allergic rhinitis
   ☐ Other (please specify)__________
3. Have you ever used one of the following prescription medication?
   ☐ Anti-hypertensives ☐ Hypolipidemic agents ☐ Painkiller
   ☐ Upset stomach relief ☐ Muscle relaxant ☐ Calcium intakes
   ☐ Hypnotics ☐ Other (please specify)__________

IV. **Complementary and alternative medicine (CAM) use to cope with stress during the COVID-19 pandemic period (Please choose the appropriate answer/s within the past three months)**

1. Nutritional Approaches
   - ☐ Herbs ☐ Probiotics ☐ Dietary supplements
   - ☐ Special diets ☐ Microbial-based therapies

2. Psychological Approaches
   - ☐ Meditation ☐ Music therapies ☐ Relaxing therapies

3. Physical Approaches
   - ☐ Acupuncture ☐ Massage ☐ Spinal manipulation

4. Combinations of Psychological and Physical or Psychological and Nutritional Approaches
   - ☐ Yoga ☐ Tai chi ☐ Qigong
   - ☐ Art therapies ☐ Aromatherapy ☐ Dance therapies
   - ☐ Mindful eating

5. Other Complementary Health Approaches
   - ☐ Traditional Chinese medicine ☐ Homeopathy
   - ☐ Naturopathy ☐ Ayurvedic medicine ☐ Functional medicine

6. Other
   - ☐ Far infrared rays ☐ Reading scriptures or bible
   - ☐ Fortune-telling
